# Segmentation Method for Ship-Radiated Noise Using the Generalized Likelihood Ratio Test on an Ordinal Pattern Distribution

**DOI:** 10.3390/e22040374

**Published:** 2020-03-25

**Authors:** Lei He, Xiao-Hong Shen, Mu-Hang Zhang, Hai-Yan Wang

**Affiliations:** 1 School of Marine Science and Technology, Northwestern Polytechnical University, Xi’an 710072, China; heleimail@mail.nwpu.edu.cn (L.H.); xhshen@nwpu.edu.cn (X.-H.S.); zhangmuhangl@gmail.com (M.-H.Z.); 2 School of Electronic Information and Artificial Intelligence, Shaanxi University of Science and Technology, Xi’an 710021, China

**Keywords:** ship-radiated noise, audio segmentation, change-point detection, ordinal pattern

## Abstract

Due to the diversity of ship-radiated noise (SRN), audio segmentation is an essential procedure in the ship statuses/categories identification. However, the existing segmentation methods are not suitable for the SRN because of the lack of prior knowledge. In this paper, by a generalized likelihood ratio (GLR) test on the ordinal pattern distribution (OPD), we proposed a segmentation criterion and introduce it into single change-point detection (SCPD) and multiple change-points detection (MCPD) for SRN. The proposed method is free from the acoustic feature extraction and the corresponding probability distribution estimation. In addition, according to the sequential structure of ordinal patterns, the OPD is efficiently estimated on a series of analysis windows. By comparison with the Bayesian Information Criterion (BIC) based segmentation method, we evaluate the performance of the proposed method on both synthetic signals and real-world SRN. The segmentation results on synthetic signals show that the proposed method estimates the number and location of the change-points more accurately. The classification results on real-world SRN show that our method obtains more distinguishable segments, which verifies its effectiveness in SRN segmentation.

## 1. Introduction

To distinguish the statuses/categories of ships according to their radiated noise, we require audio segments to be homogeneous to extract consistent acoustic features. Therefore, audio segmentation is an essential procedure to deal with the diversity of the ship-radiated noise (SRN). The primary sources of SRN diversity in the real world are as follows:

1. The SRN consists of a variety of components, including propeller noise, hydrodynamic noise, and noise from various mechanical parts radiated into the water through the hull [1].
2. The traits of the SRN relate to the propulsion devices and operating states (entering or departing a port, waiting for boarding) of ships.
3. The SRN varies while the ship is sailing nearby the hydrophone because the near field sound around the ship is not isotropic [2].
4. As the absorption coefficient changes with the distance between the hydrophone and the sound source [3], the proportion of the high-frequency and low-frequency in the SRN spectrum shifts when a ship is approaching or leaving.

The methods for audio signal segmentation are divided into two types: the model-based methods and the metric-based methods [4]. The model-based methods train a model to explore the subtle differences among the acoustic characteristics, and then determine where a segment starts and ends using the predicted class labels. However, the model-based methods are unsuitable for the SRN segmentation, as it is difficult to obtain sufficient class labels of SRN for the training of the model, neither by manual nor clustering [5]. Different from the model-based methods, the metric-based methods measure the similarity between two adjacent segments from the statistics of the acoustic features, usually with a three-stage approach: acoustic feature extraction, estimation of the probability distribution, and detection for change-points [6]. The first stage is to extract time-evolving short-term acoustic features from data frames, such as energy, zero-crossing rate (ZCR) [7], cepstrum [8] and Mel-frequency cepstrum coefficient(MFCC) [9]. The second stage is to estimate the joint probability distribution of the acoustic features. Since the difficulty of probability distribution estimation increases with the dimension of the feature vector, the metric-based method uses only the most informative features. The third stage is to establish a criterion for change-point detection, based on the estimated probability distributions of the acoustic features. The criteria proposed in recent years include the Bayesian Information Criterion (BIC) [10], the Hausdorff distance [11], the Kullback–Leibler divergence [12,13], the novelty score [14], and so on. The BIC based audio segmentation and its variations [15,16] are popular in audio segmentation because of the low calculation and high flexibility.

Although the successful applications of metric-based segmentation on many scenarios [17,18], the SRN segmentation is still challenging for reasons as follows. Firstly, the performance of the metric-based method mostly depends on the prior knowledge about the signal, such as the distinguishable acoustic features and the probability distribution they follow. However, we have little such prior knowledge about the SRN. Secondly, many hyperparameters, such as the short-term window length and the analysis window length, need to be calibrated carefully to obtain satisfactory segmentation for practical SRN. The window lengths relate to the temporal resolution of the segmentation. The shorter the window, the higher the temporal resolution of the segmentation. However, too short window length will give rise to the inconsistent estimations of the acoustic features and their probability distribution, and thus leads to unreliable change-point detection. Thirdly, in SRN, there is no structural information like words or syllables, which play a vital role in the segmentation of speech or music. In addition, the continuous changing of amplitude and spectrum also makes the SRN segmentation more difficult.

In this paper, we proposed an ordinal pattern distribution (OPD) [19,20] based segmentation method for SRN, to improve the identification performance of ship statuses/categories. The rest of this paper is organized as follows. In Section 2, the calculation of the ordinal pattern, the OPD [19,20], and the permutation entropy are reviewed. Then, we derive a segmentation criterion for single change-point detection (SCPD) by a generalized likelihood ratio (GLR) test, and extend it to MCPD with a computation-efficient OPD estimation on a series of analysis windows. In Section 3, we evaluate the performance of the proposed method on both the synthetic signals and real-world SRN by comparing it with BIC based segmentation method. Finally, we conclude the paper in Section 4.

## 2. Materials and Methodology

This section is structured as follows. In Section 2.1, we formulate the problem of audio segmentation and introduce the motivation of the proposed method. In Section 2.2, the preliminaries and estimation of OPD are reviewed. In Section 2.3, we proposed a segmentation criterion for single change-point detection by a GLR test [21] on the OPD. In Section 2.4, we provide an algorithm for multiple change-points detection using the proposed criterion and also a computation-efficient OPD estimation on a series of analysis window.

### 2.1. Problem Formulation and Motivations

We need to answer two basic questions in audio segmentation. First, is this audio signal homogeneous? Second, supposing it is non-homogenous, where does its characteristic shift? For an audio signal, we should estimate both the number of the change-points *N* and their location simultaneously. Formally, we assume the audio signal $X=\{x_{1},\cdots ,x_{T}\}$ is a piecewise stationary process, with N change-points in it. The full set of the unknown change-points is denoted as

(1) $$S_{CP}=\left\{j_{1},j_{2},\cdots ,j_{N}\right\}\;.$$

The N+1 homogeneous segments split by the change-points are represented as

(2) $$s_{k}=\left\{\begin{array}{cc} \{x_{1},\cdots ,x_{j_{1}}\}, & \mathrm{if}k=1,\\ \{x_{j_{k-1}+1},\cdots ,x_{j_{k}}\}, & \mathrm{if}k=2,\cdots ,N,\\ \{x_{j_{k}+1},\cdots ,x_{T}\}, & \mathrm{if}k=N+1. \end{array}\right.$$

Then, the audio segmentation is formulated as

(3) $$\min_{N,S_{CP}}\sum_{j\in S_{CP}}C\left(j\right),$$

where C(j) is the similarity between the segments before and after a change-point *j*. As C(j) is computed from the probability distributions of the acoustic features, it depends on different choices of the acoustic features and their probability distribution. In this study, we perform change-point detection according to the OPD of the audio signal. The motivations are as follows:

1. Ordinal patterns explore the chronological dependencies in the signal [20,22], which is helpful to distinguish detailed structures in the SRN.
2. Different from the traditional acoustic feature extraction, ordinal patterns are computed efficiently on the waveform of the signal, which supports a higher temporal resolution of change-point detection.
3. As a discrete probability distribution, the estimation of OPD is more convenient and straightforward than the probability distribution estimation in the traditional segmentation method, which requires the pre-change and post-change probability distributions to be known and has high computational cost.
4. Because nonlinear drift or amplitude scaling does not change the ordinal pattern [23], the variations in the amplitude of the SRN have little impact on the OPD. Therefore, OPD based segmentation reduces the performance deterioration when the distance and direction between the hydrophone and the ship are changing.

### 2.2. Efficient Estimation of Ordinal Pattern Distribution

The ordinal pattern is defined by the relationships among values of adjacent data points. Before the calculation of ordinal pattern, the original signal *X* is embedded with dimension *m* and time delay τ, as

(4) $$\begin{array}{cc} X_{t} & =\{x_{t},\cdots ,x_{t+(m-1)\tau }\},\\ X_{E} & =\{X_{1},\cdots ,X_{T-(m-1)\tau }\}, \end{array}$$

where *m* equals to the order of the ordinal pattern, and τ depends on the time-varying characteristics of the target signal. The embedding dimension *m* and time delay τ for a specified signal are selected heuristically by evaluating the average normalized entropy of a set of distributions [24].

According to the values in $X_{t}$, an ordinal pattern is represented by the ranking operator $r(X_{t}^{k})$ as

(5) $$\begin{array}{c} o_{t}=\left[r\left(X_{t}^{1}\right),r\left(X_{t}^{2}\right),\cdots ,r\left(X_{t}^{m}\right)\right], \end{array}$$

where $r(X_{t}^{k})$ is the the ranking index of the k-th element in $X_{t}$, and $r(X_{t}^{k})\in 1,2,\cdots ,m$. For example, if $X_{t}^{k}$ is the second largest element in $X_{t}$, then $r(X_{t}^{k})=2$. Additionally, $r\left(X_{t}^{k}\right)>r\left(X_{t}^{k+1}\right)$ if $X_{t}^{k}=X_{t}^{k+1}$.

We use Π to denote the full set of the possible ordinal patterns as

(6) $$\Pi :=\{\pi_{1},\pi_{2},\cdots ,\pi_{m!}\},$$

where each π∈Π corresponding to a specific order of the elements in $X_{t}$ [25]. Then, using Equation (5), $X_{E}$ is transformed into a sequence of π as

(7) $$X_{E}\to O_{\pi }=\left\{o_{1},\cdots ,o_{T-(m-1)\tau }\right\},$$

where $o_{t}\in \Pi$.

The OPD describes the probability of the ordinal pattern $o_{t}$ taking each possible π. The corresponding probability mass function is

(8) $$o_{t}∼O\left(p\right):p\left(o_{t}\right)=\prod_{k=1}^{K}p_{\pi_{k}}^{\left[o_{t}=\pi_{k}\right]},$$

where K=m! is the number of possible permutations, and *m* is the order of the ordinal patterns. $p_{\pi_{k}}$ represents the probability of occurrences of $\pi_{k}$ and satisfies $\sum_{k=1}^{K}p_{\pi_{k}}=1$. [] is the Iverson bracket, which takes a value of one when the condition in the parentheses is true; otherwise, it is zero.

In practical application of SRN segmentation, the procedure of mapping from $X_{E}$ to $O_{\pi }$ is computation-intensive, since the computation cost of permutation estimation is equal to that of sorting. Considering the overlaps of $X_{t}$ in $X_{E}$, we use the left inversion count *l* [26] to construct an efficient mapping function. $l_{i}\left(x\right)$ is defined as the number of elements in a sequence *x* greater than x(i) before x(i), as

(9) $$l_{i}\left(x\right)=\#\left\{k|k<i\wedge x\left(k\right)>x\left(i\right)\right\},$$

where #{} denote the number of elements in the set. According to the inversions of permutations [27], equivalent to Equation (5), the ordinal pattern can be represented with a sequence of left inversion counts, as

(10) $$o_{t}=\left[l_{1}\left(X_{t}\right),l_{2}\left(X_{t}\right),\cdots ,l_{m}\left(X_{t}\right)\right].$$

According to Equation (4), let $X_{t}^{\prime }$ denote the last m−1 elements in $X_{t}$, which are identical to the first m−1 elements in $X_{t+1}$, we compute the left inversion counts of $X_{t}^{\prime }$ as

(11) $$l_{k}\left(X_{t}^{\prime }\right)=\left\{\begin{array}{cc} l_{k}\left(X_{t}\right), & \mathrm{if}\;X_{t}\left(1\right)<X_{t}\left(l\right),\\ l_{k}\left(X_{t}\right)-1, & \mathrm{else}. \end{array}\right.$$

Then, $o_{t+1}$ is estimated from $l_{k}\left(X_{t}^{\prime }\right)$ with at most m−1 comparisons, as

(12) $$\begin{array}{cc} l_{k}\left(X_{t+1}\right) & =\left\{\begin{array}{cc} l_{k+1}\left(X_{t}^{\prime }\right), & k=1,\cdots ,m-1,\\ \#\{X_{t}^{\prime }>X_{t+1}\left(m\right)\}, & k=m, \end{array}\right.\\ o_{t+1} & =[l_{1}\left(X_{t+1}\right),l_{2}\left(X_{t+1}\right),\cdots ,l_{m}\left(X_{t+1}\right)]. \end{array}$$

**Figure 1:**
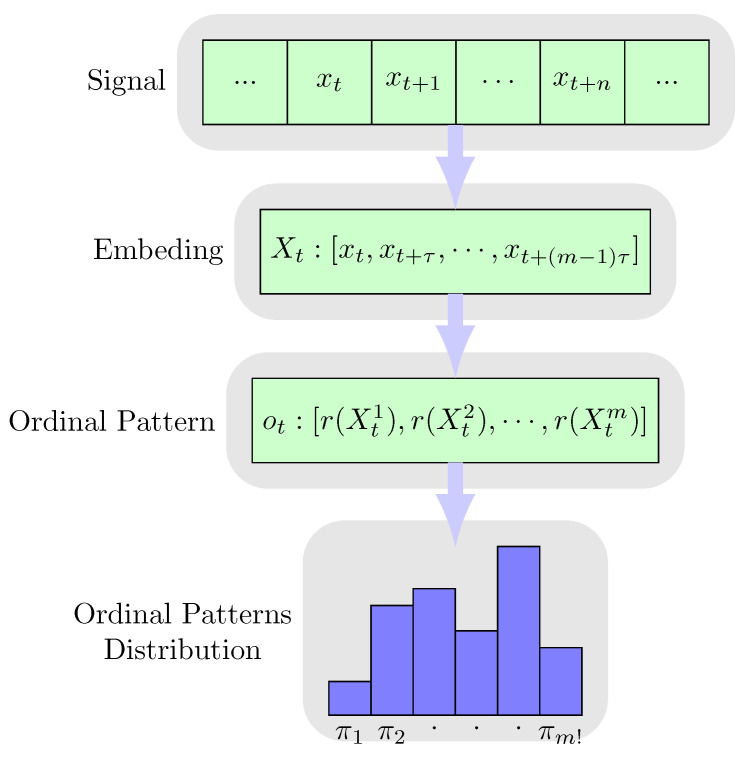
The estimation process of ordinal pattern distribution (OPD) from the audio signal.

By computing the index of π in Π, $X_{E}$ is directly mapped to $O_{\pi }$ according to

(13) $$d\left(X_{t}\right)=1+\sum_{k=1}^{m}l_{i}\left(X_{t}\right)\left(k-1\right)!,$$

where d∈{1,2,⋯,m}, and $\pi_{d(X_{t})}$ is the ordinal pattern of $X_{t}$. Finally, the coefficients in Equation (8) are estimated from the number of occurrences of each π in $O_{\pi }$, as

(14) $$p_{\pi_{k}}=\frac{\sum_{i=1}^{T-(m-1)\tau }\left[d\left(X_{t}\right)=k\right]}{T-(m-1)\tau },$$

where k=1,⋯,K. The whole process of the OPD estimation is shown in Figure 1.

### 2.3. Proposed Criterion for Single Change-Point Detection

The key ingredient of the single change-point detection is the criterion of whether a change-point exists in a signal. For simplification, we assume that there is at most one change-point in an analysis window with length *T*. $X^{0}=\left(x_{1},x_{2},\cdots ,x_{T}\right)$ is the audio signal in the analysis window. In case that a change-point *j* exists, the two segments separated by the unknown change-point *j* are

(15) $$\begin{array}{cc} X^{1} & =(x_{1},x_{2},\cdots ,x_{j}),\\ X^{2} & =(x_{j+1},x_{j+2},\cdots ,x_{T}). \end{array}$$

According to Section 2.2, $o_{t}^{0}$, $o_{t}^{1}$, and $o_{t}^{1}$ are the sequence of ordinal patterns corresponding to $X^{0}$, $X^{1}$, and $X^{2}$, respectively.

SCPD can be formulated as a hypothesis testing for model selection [28,29]. Based on the OPD, our hypothesis testing for SCPD is stated as follows. The null hypothesis $H_{0}$ states that $o_{t}^{0}$ follows the OPD $O(p^{0})$, as

(16) $$H_{0}:\;o_{t}∼O\left(p^{0}\right),\;t=1,2,\cdots ,T-\left(m-1\right)\tau ,$$

where $p^{0}=\left(p_{1}^{0},p_{2}^{0},\cdots ,p_{K}^{0}\right)$ is the parameters of the OPD, and satisfies $\sum_{i=1}^{K}p_{i}^{0}=1$. The alternative hypothesis states that $o_{t}^{1}$ and $o_{t}^{2}$ follow two OPD with distinct parameters. Under the alternative hypothesis $H_{1}$, the pre-change and post-change distribution are denoted by $O(p^{1})$ and $O(p^{2})$ as

(17) $$H_{1}:\;\left\{\begin{array}{c} o_{t}∼O\left(p^{1}\right)\;t=1,\cdots ,j-\left(m-1\right)\tau ,\\ o_{t}∼O\left(p^{2}\right)\;t=j+1,\cdots ,T-\left(m-1\right)\tau , \end{array}\right.$$

where $p^{1}$ and $p^{2}$ are the free parameters of $O(p^{1})$ and $O(p^{2})$.

Using $p^{0}$, $p^{1}$ and $p^{2}$, which are estimated efficiently by Equation (14), we compute the log-likelihood functions of the null hypothesis $H_{0}$ and the alternative hypothesis $H_{1}$ as

(18) $$\begin{array}{ccc} L_{H_{0}} & = & \sum_{t=1}^{T-(m-1)\tau }\sum_{i=1}^{K}\left[o_{t}=\pi_{i}\right]\log\left(p_{i}^{0}\right).\\ L_{H_{1}} & = & \sum_{t=1}^{j-(m-1)\tau }\sum_{i=1}^{K}\left[o_{t}=\pi_{i}\right]\log\left(p_{i}^{1}\right)\\ & & +\sum_{t=j+1}^{T-(m-1)\tau }\sum_{i=1}^{K}\left[x_{t}=i\right]\log\left(p_{i}^{2}\right). \end{array}$$

Substituting Equation (14) into Equation (18), we have

(19) $$\begin{array}{cc} L_{H_{0}} & =\left(m\tau -\tau -T\right)\cdot PE^{0},\\ L_{H_{1}} & =\left(m\tau -\tau -j\right)\cdot PE^{1}+\left(m\tau -\tau +j-T\right)\cdot PE^{2}, \end{array}$$

where $PE^{n}=-\sum_{i=1}^{K}p_{i}^{n}\log\left(p_{i}^{n}\right)$ and n=0,1,2.

According to Wilks’ theory [30], under the null hypothesis $H_{0}$, when the sample size approaches infinity, the likelihood ratio asymptotically approximates the $\chi^{2}$ distribution, and the degree of freedom equals the difference between the numbers of parameters in $H_{0}$ and $H_{1}$. Therefore, the generalized likelihood ratio of our hypothesis test is approximated as

(20) $$LR=-2\log\frac{L_{H_{0}}}{L_{H_{1}}}∼\chi^{2}\left(m!-1\right),$$

where *m* is the order of the ordinal pattern.

Then, the criterion for SCPD is established from Equation (20). As the estimation of LR varies with the location of the tentative change-point *j*, we use the maximum of LR to test the existence of the change-point. With a given significance level α, a change-point is detected if

(21) $$\max_{j\in (1,T)}LR\left(j\right)>ICDF_{\chi^{2}\left(m!-1\right)}\left(1-\alpha \right),$$

where ICDF represents the inverse cumulative distribution function of the $\chi^{2}$ distribution. The location of the detected change-point is estimated as

(22) $$\begin{array}{c} \hat{j}={\mathrm{argmax}}_{j}LR\left(j\right). \end{array}$$

Conversely, we reject the existence of a change-point if Equation (21) does not hold.

### 2.4. Computation-Efficient Multiple Change-Points Detection with a Variable Window

Audio segmentation is essentially an MCPD in practice. MCPD is more challenging than SCPD, as its main goal is to estimate the number and location of change-points simultaneously, which means exploring an ample segmentation space. Therefore, the calculation cost of the MCPD algorithm increases with the number of data points. The pros and cons of many MCPD algorithms have been reviewed in [31], including exhaustive search, stepwise selection, L1 penalization, and so on. Computation cost is an important consideration in ship-radiated noise processing because the data points per second are much more than that of the signals from physical dynamics or the economic process. In this section, we extend the proposed SCPD to the multiple change-points case and reduce the computation cost taking advantage of the sequential structure of the OPD.

We assume that the audio signal follows a piecewise stationary model, with an unknown number of change-points in the OPD. Under this assumption, by testing each data point as a candidate change-point, we generalize the hypothesis testing for a single change-point detection to the multiple change-points detection. The null hypothesis $H_{0}$ states that data point *j* is a change-point, while the alternative hypothesis states that the data point *j* is not a change-point. Obviously, for an audio signal, there is a large number of tests. In addition, as these tests follow a sequential structure, they do not belong to the typical multiple testing [32]. According to the sequential structure, the test is performed in a series of analysis windows, as

(23) $$\begin{array}{cc} H_{0} & :\;o_{t}∼O\left(p^{0}\right),\;t\in \left[j-L,j+L\right],\\ H_{1} & :\;\left\{\begin{array}{c} o_{t}∼O\left(p^{1}\right)\;t\in \left[j-L,j\right),\\ o_{t}∼O\left(p^{2}\right)\;t\in \left[j,j+L\right], \end{array}\right. \end{array}$$

where *L* is the the length of the analysis window, $p^{1}$, and $p^{2}$ are the parameters of the two different OPDs, and $p^{1}\neq p^{2}$. Using Equation (23), we can efficiently estimate the OPDs over shifting windows in two steps. First, to avoid redundant hashing of an ordinal pattern from the same location, the hashing result at each data point is stored for repeated use in tests on different analysis windows. Then, the OPD is estimated in a manner similar to CUMSUM in [33,34] but very straightforward. Specifically, by computing the cumulative sum of the number of occurrences for each ordinal pattern point by point, the OPD is estimated from the difference between the two cumulative sums at the beginning and end of the analysis window, instead of counting the occurrences of each ordinal pattern in the analysis window. In this way, we obtain the number of occurrences for each ordinal pattern in each possible segmentation efficiently. Then, the cumulative sum of the number of occurrences for each ordinal pattern at time *t* is

(24) $$Cum_{t}=\left\{C_{k,t}\right\},k=1,2,\cdots ,m!,$$

where $C_{k,t}$ represents the number of occurrences for the ordinal pattern $\pi_{k}$ during time 1-*t*. $C_{k,t}$ can be computed in an iterative manner, as

(25) $$\begin{array}{cc} C_{k,1} & =[o_{1}=\pi_{k}],\\ C_{k,t+1} & =C_{k,t}+\left[o_{t+1}=\pi_{k}\right], \end{array}$$

where $o_{t}\in O_{\pi }$. Then, the corresponding permutation entropy $PE(t_{1},t_{2})$ of analysis window $\left(t_{1},t_{2}\right]$ from the difference of $Cum_{t_{1}}$ and $Cum_{t_{2}}$ is

(26) $$\begin{array}{cc} \Delta_{Cum} & =Cum_{t_{2}}-Cum_{t_{1}}=\left[\delta_{1},\delta_{2},\cdots ,\delta_{m!}\right],\\ p_{\pi_{k}} & =\frac{\delta_{k}}{\sum_{i=1}^{m!}\delta_{i}},\\ PE(t_{1},t_{2}) & =-\sum_{k=1}^{m!}p_{\pi_{k}}\log p_{\pi_{k}}. \end{array}$$

**Figure 2:**
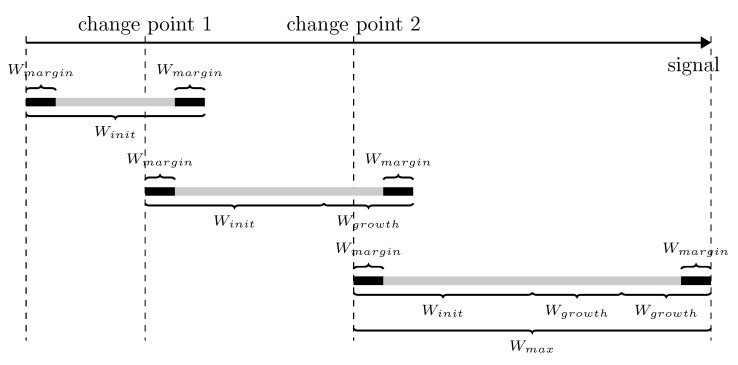
The local search strategy with a variable analysis window.

An essential characteristic of the multiple change-points detection is its local nature. The OPD of an audio segment depends only on the ordinal patterns in the range from the previous change-point to the next change-point. The estimated distribution might be biased due to the use of ordinal patterns outside the specific range, which follows different probability distributions. Therefore, a shifting analysis window with fixed length as Equation (23) may deteriorate the segmentation performance. However, as the next change-point of the data point under test is unknown, it is infeasible to use only the ordinal patterns relevant to the current hypothesis testing. Considering the local nature of the multiple change-point detection, we use a searching strategy for change-point with a variable analysis window. At the beginning, we check whether there is a change-point in an analysis window of length $W_{init}$. If a change-point exists, the precise location of the change-point is then estimated by Equation (21). Correspondingly, if no change-point detected, we grow the length of the analysis window in steps of $W_{grow}$ until it contains a change-point or reaches the maximum window length $W_{max}$. After a successful detection of a change-point, we perform SCPD in a new analysis window of length $W_{init}$ begin from the detected change-point. If the length of the analysis window reaches the maximum with no change-point detected, we begin a new SCPD from the end of the previous analysis window. We repeat the test and obtain multiple change-points sequentially until the analysis window reaches the end of the signal. In Figure 2, we illustrate the local search strategy with a variable window.

Another important consideration is that there exist many types of random noise with unknown distribution in the SRN. It is easy to detect false change-points in the region where the signal-to-noise ratio is low [35]. The false change-points will result in many additional small segments in the audio segmentation, which is prone to inducing errors in the processing of the SRN. Following [36], we add a minimum length constraint $W_{margin}$ in the proposed method for the MCPD. With the significance level α and a variable analysis window, the proposed method is illustrated in Algorithm 1.
**Algorithm 1** MCPD with a variable window**Require:**$X,W_{init},W_{grow},W_{max},W_{margin},\alpha$1:Calculate $O_{\pi },Cum_{t}$ from Equation (7), (24), and (25)2:CP=∅3:$t_{1}=0,t_{2}=W_{init}$4:**while**$t_{2}\leq$ length(*X*) **do**5:    **for** $j=t_{1}+W_{margin}:t_{2}-W_{margin}$ **do**6:        Calculate $L_{H_{0}}$ and $L_{H_{1}}$ from Equation (19)7:        Calculate LR(j) from Equation (20)8:    **end for**9:    $j_{candidate}=\mathrm{argmax}\left(LR\right)$10:    $LR_{max}=LR\left(j_{candidate}\right)$11:    **if** $LR_{max}<ICDF_{\chi^{2}\left(m!-1\right)}\left(1-\alpha \right)$ **then**12:        **if** $t_{2}-t_{1}<W_{max}$ **then**13:           $t_{2}=t_{2}+W_{grow}$14:        **else**15:           $t_{1}=t_{2}$16:           $t_{2}=t_{2}+W_{init}$17:        **end if**18:    **else**19:        $CP=CP\cup j_{candidate}$20:        $t_{1}=j_{candidate}+1$21:        $t_{2}=t_{1}+W_{init}$22:    **end if**23:**end while**

## 3. Results and Discussion

In this section, we evaluate the performance of the proposed method on both synthetic signals and real-world SRN, by comparison with the BIC based segmentation methods [10].

The BIC based algorithm is the most widely used method in audio segmentation. Because increasing the number of model parameters improves the likelihood function but makes the model prone to overfit, the BIC method introduces a penalty factor λ related to the number of model parameters in the likelihood function, as

(27) $$BIC\left(M\right)=\log L\left(X,M\right)-\frac{1}{2}\lambda m\log\left(N\right),$$

where *L* is the likelihood function, *X* is the set of samples, *M* is a parametric model, *m* is the number of free parameters in the model, and *N* is the number of samples. According to Equation (27), we transform the audio segmentation into a model selection problem. In the case that no change-point exists in the sequence of short-term acoustic features, we use $M_{0}$ to model the statistical characteristics of the acoustic features in the analysis window. In the case that a change-point exists in the sequence of short-term acoustic features, we model the statistical characteristics of the acoustic features in the segments before and after the change-point with $M_{1}$ and $M_{2}$, respectively. Then, the log-likelihood ratio of the two cases is

(28) $$LR\left(i\right)=BIC\left(M_{0}\right)-BIC\left(M_{1}\right)-BIC\left(M_{2}\right).$$

If LR(i)>0, the estimated change-point is located where the LR(i) reaches the maximum value. More detailed information about the BIC audio segmentation can be referred to in [10].

To make a fair comparison for MCPD, the BIC audio segmentation method and the proposed method share the same search strategy and hyperparameters. In addition, as the SRN varies mainly in amplitude and frequency, we choose the energy and the ZCR as the basic acoustic features. Then, we use the normal distribution to model the statistics of the acoustic features. In the following, we refer to the BIC based segmentation methods on the energy and the ZCR as the BSoE and the BSoZ, respectively. Compared to the ordinal pattern, the estimation of these two acoustic features requires a longer short-term window $W_{short}$. We set the short-time window length $W_{short}$ as 50 to compute short-term acoustic features. Additionally, we set the step size of the window to one, so that the temporal resolution of the three methods are all one.

This section is organized as follows. In Section 3.1, we conduct experiments on synthetic signals. The performance of the proposed method is evaluated on three different types of signals that are generated, including signals with single change-point, signals without change-point, and signals with multiple change-points. In Section 3.2, we apply the proposed method on the ShipsEar dataset [37], and measure the segmentation performance by time-weighted classification accuracy of the segments.

### 3.1. Segmentation of the Synthetic Signal

#### 3.1.1. Single Change-Point Detection

We generate three different types of signals to evaluate the performance of the proposed method for SCPD, namely $y_{mix}$, $y_{N}$, and $y_{chirp}$. $y_{mix}$ consists of two different parts. The first part of $y_{mix}$ is Gaussian white noise, and the second part of $y_{mix}$ is a single-frequency signal contaminated by Gaussian white noise. The formula to generate $y_{mix}$ is

(29) $$y_{mix}=\left\{\begin{array}{cc} n_{1}\left(t\right), & \;\mathrm{if}\;t=1,\cdots ,j,\\ \sqrt{3}\sin\left(0.2\pi t\right)+n_{2}\left(0,\frac{3}{20}\right), & \;\mathrm{if}\;t=j+1,\cdots ,T, \end{array}\right.$$

where $n_{1}\left(t\right)∼N\left(0,1\right)$ and $n_{2}\left(t\right)∼N\left(0,\frac{3}{20}\right)$ are Gaussian white noise [38]. According to the coefficients in Equation (29), the power ratio of the first part to the latter one is 2:3, and, in the second part, the power ratio of the single-frequency signal to the random noise is 10:1. $y_{N}$ is Gaussian white noise with gradually increasing amplitude, and $y_{chirp}$ is a chirp signal with gradually increasing frequency. The formulas to generate $y_{N}$ and $y_{chirp}$ are

(30) $$\begin{array}{cc} y_{N} & =\left(\frac{t}{T}+0.5\right)n\left(t\right),\\ y_{chirp} & =\sin\left(2\pi (0.1+\frac{0.02t}{T})\right), \end{array}$$

where n(t)∼N(0,1) and t=1,2,⋯,T.

**Figure 3:**
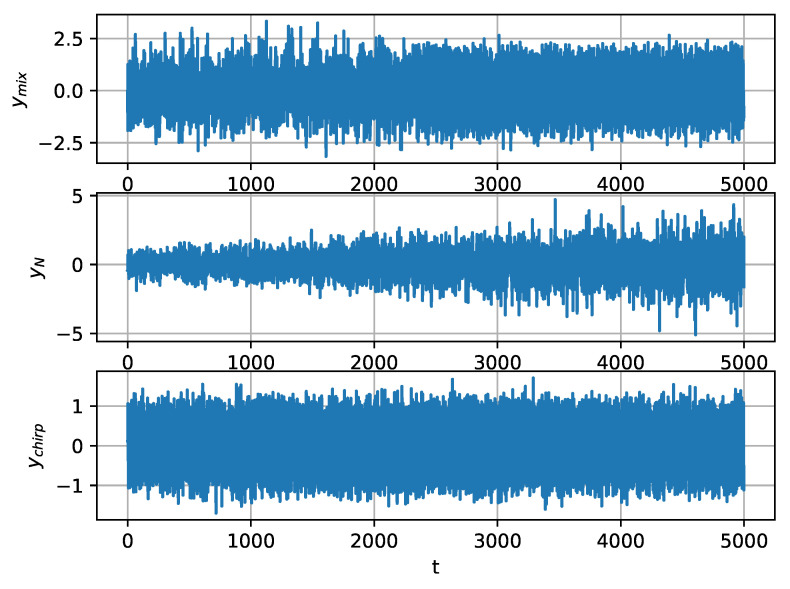
Synthetic signals generated for performance evaluation for single change-point detection (SCPD).

In these experiments, $y_{mix}$ approximates the SRN with a line spectrum submerged in ambient noise, $y_{N}$ approximates the ambient noise with gradually increasing amplitude, and $y_{chirp}$ approximates the SRN generated from a propeller whose rotation speed is increasing. In the latter two cases, no change-point exists in the signal. The three types of synthetic signals are shown in Figure 3.

**Figure 4:**
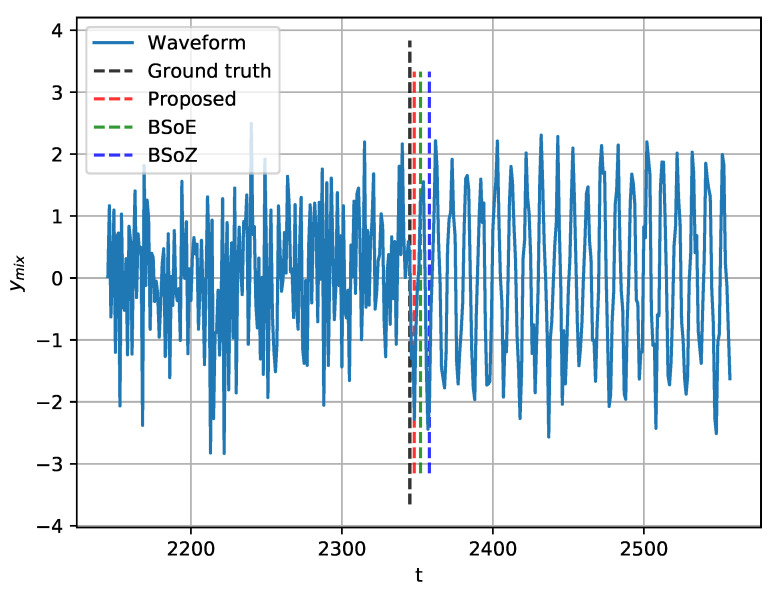
Single change-point detection (SCPD) on a random realization of $y_{mix}$, with true change-point at j=2345.

**Figure 5:**
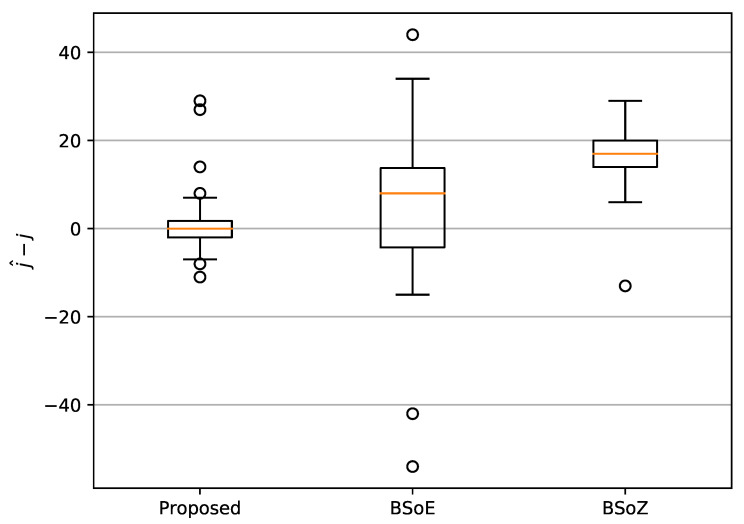
The box-and-whisker plots of the change-point estimation bias of the three methods.

Figure 4 shows the results of SCPD on a random realization of $y_{mix}$, which has a change-point at j=2345. Though the locations of the estimated change-points vary from each other, all three of the methods achieve a satisfactory accuracy. Furthermore, to lower the bias due to the random realization of $y_{mix}$, we generate 50 different realizations of $y_{mix}$ with change-points randomly located in the range (500, 4500). The performance of the three methods for SCPD is measured by $j-\hat{j}$, the distance of the estimated change-point from the true change-point. The distributions of $j-\hat{j}$ are shown by the box-and-whisker plots in Figure 5.

As shown in Figure 5, the mean of $j-\hat{j}$ computed by our method is approximately 0, while that of the BSoE and the BSoZ are about five and 15, respectively. The comparison of the mean of $j-\hat{j}$ indicates that our algorithm detected change-point with more precise locations. One explanation for this is that both the BSoE and the BSoZ require a short-term window $W_{short}$ to calculate the acoustic features. Instead, the proposed method computes ordinal patterns from only *m* data points on the waveform. As $m\ll W_{short}$, our method has a higher temporal resolution. In addition, Figure 5 also shows that the proposed method has the smallest standard deviation of $j-\hat{j}$, which indicates the robustness of the proposed method.

**Figure 6:**
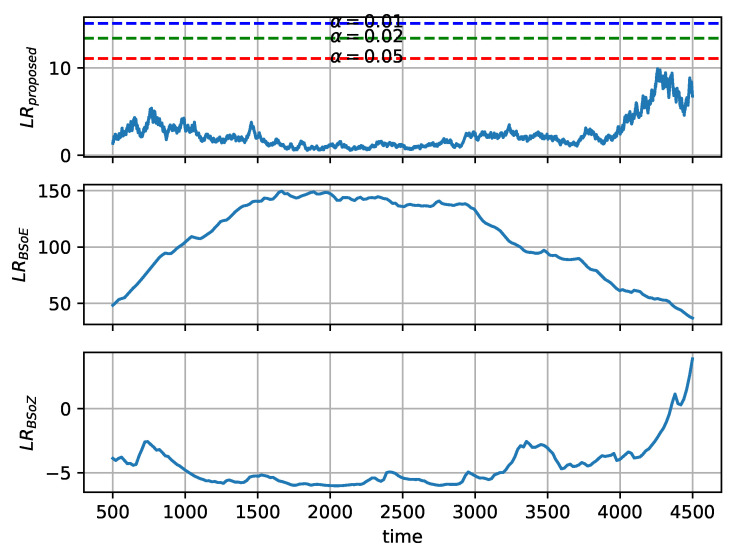
Evaluation of the proposed methods, the bayesian information criterion (BIC) based segmentation on energy (BSoE), and the BIC based segmentation on zero-crossing rate (BSoZ) for segmentation of $y_{N}$.

**Figure 7:**
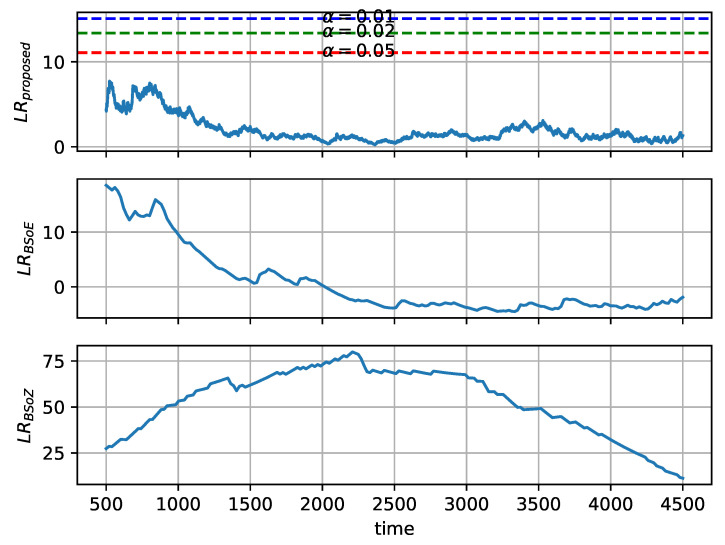
Evaluation of the proposed methods, the bayesian information criterion (BIC) based segmentation on energy (BSoE), and the BIC based segmentation on zero-crossing rate (BSoZ) for segmentation of $y_{chirp}$.

As there is no change-point in $y_{N}$ or $y_{chirp}$, we investigate the log-likelihood ratio ($LR_{proposed},LR_{BSoE},LR_{BSoZ}$) of the hypothesis test. The log-likelihood ratio at each data point in $y_{N}$ and $y_{chirp}$ is shown in Figure 6 and Figure 7, respectively. The existence of change-point is tested by whether the log-likelihood ratio exceeds the corresponding critical threshold. According to Equation (21), the critical threshold of the proposed method relies on the significance level α. Three critical thresholds are shown in Figure 6 and Figure 7, corresponding to α=0.01, α=0.02, and α=0.05. According to Equation (28), the thresholds of the BSoE and the BSoZ are both 0.

Figure 6 shows that the BSoE detects a change-point in the middle of $y_{N}$, whereas the BSoZ reports no change-point because the frequency of $y_{N}$ does not change over time. The log-likelihood ratio of the BSoZ on $y_{N}$ is generally below zero, except that a false change-point detected at the end of $y_{N}$, where $LR_{BSoZ}>0$. As shown in Figure 7, the BSoZ reports a change-point in the middle of $y_{chirp}$, where the BSoE detects no change-point as the log-likelihood ratio is below zero. In addition, the BSoE overestimates the log-likelihood ratio at the beginning of $y_{chirp}$.

Overall, the BSoE estimates a change-point in the middle of $y_{N}$, while the BSoZ locates a change-point in the middle of $y_{chirp}$. Both the BSoE and the BSoZ overestimate the log-likelihood ratio at the beginning or end of the signal because the length of the two tentative segments is significantly different. Since the mean and the variance among neighboring data points are not considered in ordinal pattern analysis [23], the proposed method detects no change-point on both $y_{N}$ and $y_{chirp}$, which is favorable in SRN segmentation. Additionally, compared with the BSoE and the BSoZ, the log-likelihood ratio of our method exhibits smaller deviations, which implies its robustness to random noise.

#### 3.1.2. Multiple Change-Points Detection

**Figure 8:**
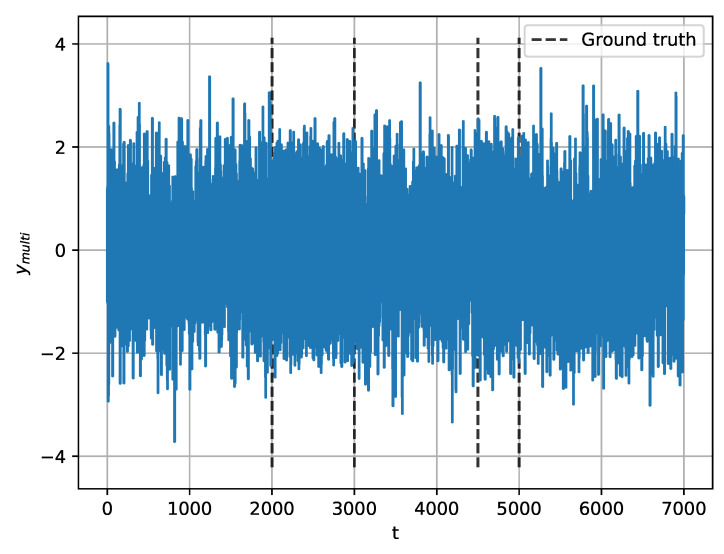
Synthetic signal for multiple change-points detection (MCPD).

In this section, we investigate the performance of the proposed method for MCPD. Using Equation (31), we generate synthetic signals with four change-points at 2000, 3000, 4500, and 5000, as shown in Figure 8:

(31) $$y_{multi}=\left\{\begin{array}{cc} N(0,1), & \;\mathrm{if}\;t=1,\cdots ,2000,\\ \sqrt{3}\sin\left(0.2\pi t\right)+N\left(0,\frac{3}{20}\right) & \;\mathrm{if}\;t=2001,\cdots ,3000,\\ N(0,1), & \;\mathrm{if}\;t=3001,\cdots ,4500,\\ \sqrt{3}\sin\left(0.2\pi t\right)+N\left(0,\frac{3}{20}\right) & \;\mathrm{if}\;t=4501,\cdots ,5000,\\ N(0,1), & \;\mathrm{if}\;t=5001,\cdots ,7000. \end{array}\right.$$

We establish three metrics, $e_{num}$, $\bar{e}$ and $e_{max}$, to measure the performance of the three methods for MCPD. As false change-points are inevitable in the presence of noise [39], the estimated change-points do not correspond one-to-one with the true change-points. In this experiment, for a true change-point $p^{t}$, the estimated change-point closest to it is selected as its estimation, denoted as $p^{r}$. The performance metrics are calculated according to

(32a) $$\begin{array}{cc} e_{num} & =N_{e}-N_{p}, \end{array}$$

(32b) $$\begin{array}{cc} \bar{e} & =\frac{1}{N_{p}}\sum_{l=1}^{N_{p}}\left|p_{l}^{t}-p_{l}^{r}\right|, \end{array}$$

(32c) $$\begin{array}{cc} e_{max} & =\max(|p_{l}^{t}-p_{l}^{r}\left|\right),l=1,\cdots ,N_{p}, \end{array}$$

where $N_{p}$ is the number of true change-points, $N_{e}$ is the number of estimated change-points, and $e_{num}$ is the difference between $N_{e}$ and $N_{p}$. $\bar{e}$ and $e_{max}$ are the mean and maximum of the bias $|p^{t}-p^{r}|$, respectively. $e_{num}$ reflects the robustness of MCPD, and $\bar{e}$ and $e_{max}$ measure the accuracy of the estimated change-points collectively.

The three methods are tested multiple times with different initial window length $W_{init}$ and window growth length $W_{grow}$, in order to evaluate their performance for MCPD. In addition, in the test on a realization of $y_{multi}$, the three methods share the same $W_{init}$ and $W_{grow}$. According to the assumption in SCPD, $W_{init}$ is chosen to avoid the two nearest change-points (j=4500 and j=5000) included in one analysis window. Specifically, for $y_{multi}$, the critical value of $W_{init}$ is 2000. Five different $W_{init}$ are used in the tests, i.e., 500, 1000, 1500, 2000, 2500, and the corresponding $W_{grow}$ are set as 1/4 or 1/2 of the $W_{init}$. For each combination of $W_{init}$ and $W_{grow}$, we apply the three methods on 50 random realizations of $y_{multi}$ and list the performance metrics in Table 1.

The $e_{num}$ in Table 1 indicates the accuracy of the estimated change-point number. When $W_{init}$ is below the critical value, the larger the value it takes, the smaller the $e_{num}$. In the case $W_{init}$ equals 2000, the $e_{num}$ of all the three methods reach the minimum. In addition, the proposed method achieves the least $e_{num}$, about 1/10 of the other two. With $W_{init}$ below 2000, the $e_{num}$ of the proposed method is significantly smaller than that of the BSoE and the BSoZ. If $W_{init}$ violates the assumption in SCPD ($W_{init}=2500$), the $e_{num}$ of the proposed method is larger than that of the other two methods. Additionally, $W_{grow}$ also affects the results of MCPD, but only as a fine-tuning of $W_{init}$.

The $\bar{e}$ and $e_{max}$ in Table 1 measure the accuracy of the estimated change-point location collectively. The $\bar{e}$ of the BSoE and the BSoZ are small in the case that $W_{init}=500$ or $W_{init}=1000$. This is because there are more extra change-points in the results, which may decrease $\bar{e}$ and $e_{max}$, according to Equation (Section 3.1.2). The $\bar{e}$ and $e_{max}$ of all the three methods become significantly large when $W_{init}$ is above the critical value ($W_{init}=2500$). When $W_{init}$ takes other values, it has no noticeable effect on $\bar{e}$ and $e_{max}$. In the cases that $W_{init}$ follows the assumption in SCPD, $\bar{e}$ and $e_{max}$ of the proposed method are the smallest.

Overall, the results in Table 1 verify the effectiveness of our method on detect multiple change-points, even with the not well-tuned parameters.

### 3.2. Real-World Application on Ship-Radiated Noise

In this section, we apply the proposed method on the ShipsEar dataset [37], recorded in or near the port of Vigo of the Spanish Atlantic coast in northwest Spain. The sampling frequency of the hydrophones is 52,734 Hz. A high-pass filter with cut-off frequency 100 Hz was used to minimize the ambient noise in shallow water. Each record preserves different operating states as possible, such as the beginning and the end. After the removal of invalid records, the final dataset includes 90 WAV files, with length ranging from 15 s to 10 min. There are 11 different ship types and ambient noise, such as fishing boats, ocean liners, containers, and ro–ro vessels. We suggest to refer to [37] for further detailed information about this dataset.

**Figure 9:**
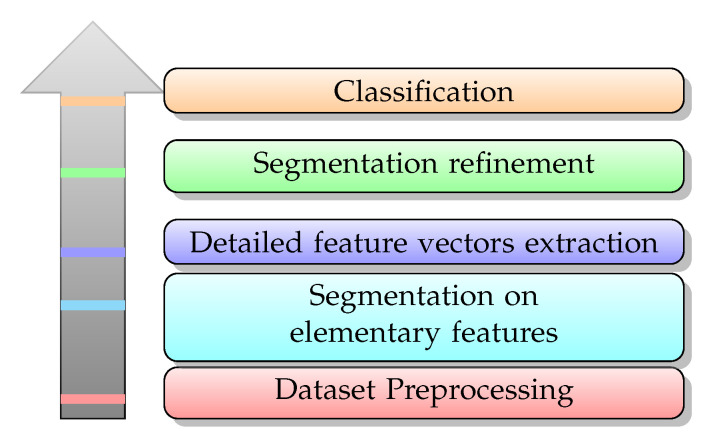
Primary stages of segmentation and classification of ship-radiated noise (SRN).

Without known annotation of the ship statuses, the segmentation performance can not be measured by the actual locations of the change-points. Therefore, we instead use the classification accuracy of the segments split by the estimated change-points. Figure 9 shows the overall flow of the segmentation and classification on SRN.

As the spectra of the SRN are concentrated in a range of low frequencies, we downsample the original signal to 6000 Hz in the preprocessing stage. With a lower sample rate, the computation cost for subsequent audio segmentation and classification is reduced while the informative characteristics of the SRN are preserved. Then, the original ship types are combined into four classes based on their tonnage [37], as the record number of some ship class is far fewer. For instance, there are only one and two records for the Trawler and the Tugboat, respectively.

In the segmentation on elementary features, we apply the BSoE, the BSoZ, and the proposed method on the SRN. Both the short-term window length and hop length in the BSoE and the BSoZ are 0.1 s. There is no overlap between adjacent windows as the window length equals the hop length. Both the energy and ZCR are computed from a window with 600 data points. In the proposed method, ordinal patterns are computed with order m=3 and time delay τ=1, according to [24]. In addition, all the parameters for MCPD in the three methods are set to the same value. Specifically, $W_{init}$ is 10 s, $W_{step}$ is 2.5 s, $W_{margin}$ is 2 s, and $W_{max}=\infty$. $W_{max}=\infty$ means that, by continuously increasing the length of the analysis window, the search for the next change-point restarts only when a valid change-point is found.

**Figure 10:**
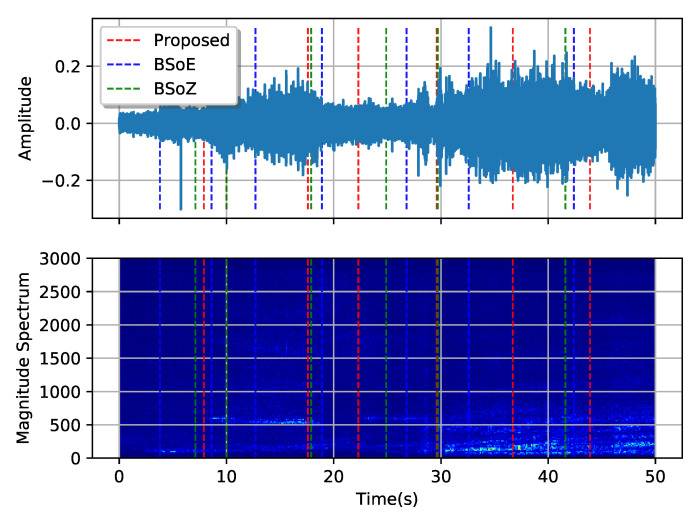
Segmentation result of a sample audio of a passenger ship entering the port.

**Figure 11:**
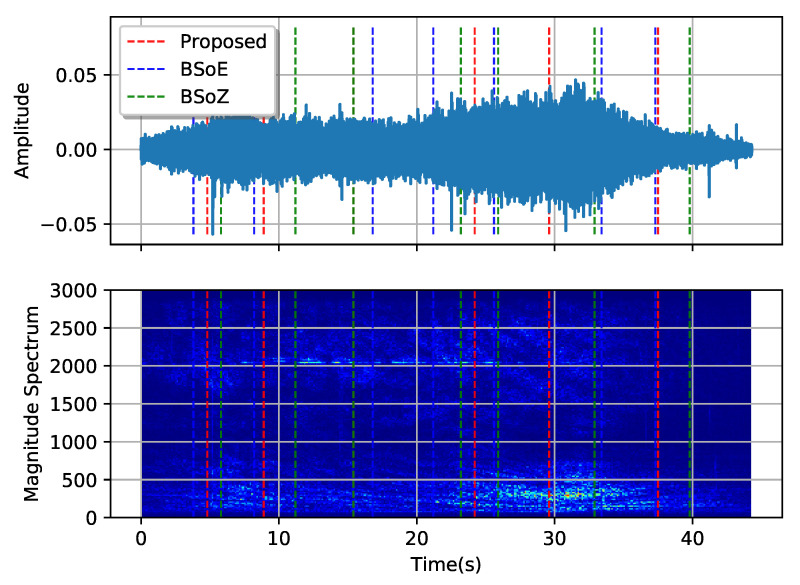
Segmentation result of a sample audio of a pilot ship passing by.

Figure 10 and Figure 11, respectively, show the results of MCPD conducted on the two typical SRN records. The first record comes from a passenger ship entering the port, and the second comes from a pilot boat passing by the hydrophone. The spectrums are calculated from the amplitudes of the short-time Fourier transform, with a short-term window length 512 sampling intervals and a hop length 256 sampling intervals. Figure 10 and Figure 11 show both the waveform and the corresponding spectrum of the two records, describing the signal from aspects of the time domain and frequency domain. The dotted lines show the locations of the estimated change-points. Shifts in both the waveforms and the corresponding spectrums of the two records are evident, but no apparent change-point exists. The three methods obtain different change-points using distinct elementary features and criteria. The BSoE tends to detect a change-point where the amplitude changes while the BSoZ inclines to report a change-point where the spectrum changes. Although the proposed method does not make use of the amplitude and frequency of the signal, it also obtains satisfactory segmentation results according to the OPD.

In the detailed feature vectors’ extraction, we extract feature vectors from the obtained audio segments using a two-stage feature extraction approach [40]. Firstly, we split each audio segment into short-term parts with equal length, and calculate acoustic features from each part. Then, we compute the statistics of the acoustic features and combine them into a detailed feature vector. Specifically, with non-overlap short-term windows of length 50 ms, we extracted a set of acoustic features using the Librosa toolkit [41], including the shape characteristics of the spectrum (centroid, bandwidth, contrast, flatness, and roll-off), the second-order polynomial coefficients of the spectrum, MFCCs, and the chroma features. There is a total of 39 elements in the acoustic features of an audio segment. For every element of the acoustic features, we calculate its mean and standard deviation and combine them into a detailed feature vector of dimension 78. Finally, these detailed feature vectors are constructed as a dataset for SRN classification.

**Figure 12:**
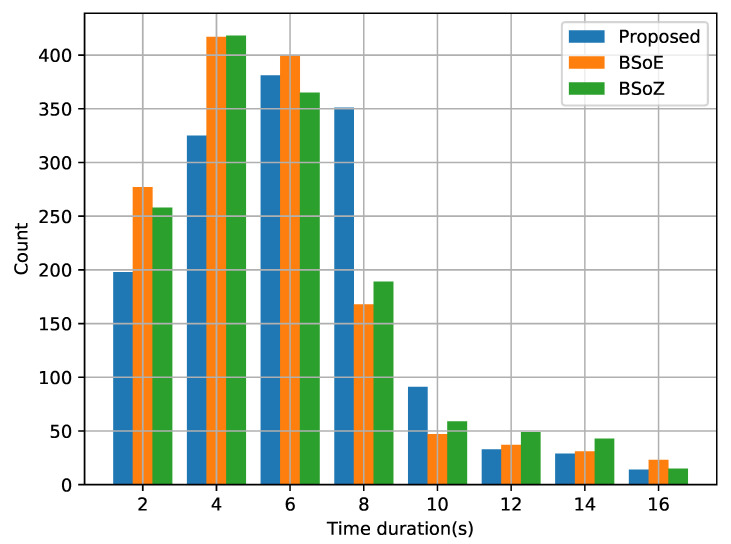
Time duration histogram of audio segments obtained by the three algorithms.

Because the audio segmentation bases only on several primary metrics, there may exist some false change-points. Therefore, we refine the result of audio segmentation according to the similarity among detailed feature vectors. In this experiment, the similarity between adjacent samples is measured by the Euclidean distance between corresponding feature vectors. If the Euclidean distance below a specified threshold, we regard the change-point between them as a false change-point and merge the two audio segments into one. In this way, we gradually increase the threshold until we obtain 1500 samples from the result of each method. Figure 12 shows the time durations of the obtained refined segments. The width of each bin in the histogram is two seconds. The segments longer than 18 s are not included in the histograms because the corresponding counts are relatively few. The time duration of the segments obtained by the proposed method mostly range from three seconds to nine seconds. In addition, compared with the other two approaches, the proposed method obtains fewer irregular audio segments.

We use two classifiers in the classification: the support vector classifier (SVC) and the random forest (RF) classifier. The two classifiers generally achieve high performance on most types of data, which is favorable to assess the quality of the obtained segments. For the SVC, we choose radial basis function as the kernel function, with regularization parameter C=1 and kernel coefficient $\gamma =1/n_{f}$, where $n_{f}=78$ is the dimension of the sample, as mentioned before. For the RF, we use Gini impurity as the indicator in the partitioning, and each RF contains 100 decision trees.

Because the time duration of the samples varies in a wide range, the classification accuracy is weighted by the time duration of the segments, which presents the time proportion of correctly classified segments in the total signal. In addition, we use stratified 10-fold cross-validation to assess the classification accuracy. Table 2 shows the weighted classification accuracy of the segments obtained by the three methods. The proposed method achieves the highest classification accuracy, either with the SVC or the RF classifier. The comparison of the classification accuracy implies that the proposed method obtained higher-quality segments of SRN. Additionally, the standard deviations of the classification accuracies obtained by the proposed method are small, which means the proposed method generates few irregular segments from SRN.

## 4. Conclusions

In this paper, we propose an audio segmentation method for SRN to improve the identification performance of ship statuses/categories. Based on the OPD, we establish a criterion for change-point detection and apply it to the SCPD and the MCPD. By comparison with the BSoE and the BSoZ, we evaluate the performance of the proposed method on both synthetic signals and real-world SRN. For the synthetic signals, the evaluation results show that the proposed method estimates the number and location of the change-points more accurately. For the real-world SRN, according to the classification results obtained by SVC and RF, the proposed method achieves the highest mean classification accuracy with a small standard deviation, which verifies the effectiveness of the proposed segmentation method.

The advantages of the proposed segmentation method for SRN are summarized as follows:

1. Using OPD as the basis for segmentation, the proposed method is free from the acoustic feature extraction and the corresponding joint probability distribution estimation.
2. As the ordinal pattern is insensitive to nonlinear drift or amplitude scaling, the proposed method reduces the number of false change-points caused by the changing distance between the ship and the hydrophone.
3. The proposed segmentation method achieves a high temporal resolution as the original pattern is calculated directly from a few data points on the signal waveform.
4. According to the sequential structure of ordinal patterns, the proposed method can efficiently estimate the OPD on a series of analysis windows, which make it applicable to real-world SRN segmentation where a large amount of data are processed.

## Figures and Tables

**Table 1:** Performance comparison for multiple change-points detection (MCPD) on multiple realizations of $y_{multi}$. The performance measures using the best parameter settings for each method are shown in bold.

Method	$W_{\mathrm{init}}$	$W_{\mathrm{grow}}$	$e_{\mathrm{num}}$	$\bar{e}$	$e_{\max}$
Proposed	500	125	3.28±1.44	8.85±4.71	21.30±12.85
Proposed	500	250	1.64±1.47	6.33±4.38	14.9±13.73
Proposed	1000	250	0.68±0.89	6.88±4.75	15±13.6
Proposed	1000	500	0.54±0.7	5.55±8.74	18.4±32.76
Proposed	1500	375	0.38±0.6	8.45±11.06	20.22±43.4
Proposed	1500	750	0.24±0.55	7.05±11.56	18.5±44.62
Proposed	2000	500	**0.02±0.14**	**6.3±14.78**	**20.6±58.15**
Proposed	2000	1000	0.1±0.3	6.35±15.46	20.8±61.18
Proposed	2500	625	−0.6±0.49	82.55±55.41	318.2±222.29
Proposed	2500	1250	−0.72±0.45	93.15±53.87	366.2±213.87
BSoE	500	125	22.44±2.23	13.85±7.07	27.2±19.8
BSoE	500	250	22.2±1.83	14.25±6.27	30±18.76
BSoE	1000	250	8.32±1.38	19±18.4	42.2±52.47
BSoE	1000	500	8.42±1.47	22.25±23.34	58.6±86.19
BSoE	1500	375	3.56±0.98	42.7±42.04	140±160.11
BSoE	1500	750	3.5±1.12	46.8±44.32	139.4±140.21
BSoE	2000	500	**1.54±0.61**	**25.5±34.64**	**75.6±122.25**
BSoE	2000	1000	1.58±0.6	26.5±31.47	76.4±105.6
BSoE	2500	625	−0.18±0.38	44.0±48.33	143.6±182.25
BSoE	2500	1250	−0.14±0.35	42.8±47.58	133.2±177.25
BSoZ	500	125	21.82±2.6	16.76±6.2	27.84±17.65
BSoZ	500	250	21.14±1.96	16±5.4	26.3±13.78
BSoZ	1000	250	8.14±1.39	19.8±10.44	41.4±38.94
BSoZ	1000	500	8.22±1.19	17.5±12.36	32±35.83
BSoZ	1500	375	3.48±0.81	32.15±27.44	91±107.28
BSoZ	1500	750	3.68±0.97	37.8±33.01	106.6±125.82
BSoZ	2000	500	**1.72±0.49**	**33.1±35.45**	**89.0±127.8**
BSoZ	2000	1000	1.82±0.38	31.3±30.21	81.8±120.97
BSoZ	2500	625	−0.02±0.14	15.6±16.81	33.6±67.07
BSoZ	2500	1250	−0.04±0.2	21.2±26.27	51.6±98.09

**Table 2:** Classification accuracy estimated by 10-fold cross-validation on the obtained ship-radiated noise (SRN) segments. The best values of the classification accuracy are shown in bold.

Method	SVC (%)	RF (%)
Proposed	**86.30 ± 4.63**	**82.71 ± 3.52**
BSoE	79.27 ± 3.49	73.60 ± 6.08
BSoZ	82.21 ± 5.37	77.75 ± 4.13

## Abbreviations

The following abbreviations are used in this manuscript:

BIC	Bayesian information criterion
BSoE	BIC based segmentation on energy
BSoZ	BIC based segmentation on zero-crossing rate
GLR	Generalized likelihood ratio
MCPD	Multiple change-points detection
MFCC	Mel-frequency cepstrum coefficient
OPD	Ordinal pattern distribution
RF	Random forest
SCPD	Single change-point detection
SRN	Ship-radiated noise
SVC	Support vector classifier
ZCR	Zero-crossing rate
